# Self-Reported Physical Inactivity and Mood Disturbances in End-Stage Kidney Disease (ESKD) Patients on Chronic Dialysis Treatment

**DOI:** 10.3390/jcm12227160

**Published:** 2023-11-18

**Authors:** Michela Musolino, Pierangela Presta, Paola Cianfrone, Maria Antonietta Errante, Michele Andreucci, Giuseppe Coppolino, Davide Bolignano

**Affiliations:** 1 Nephrology and Dialysis Unit, University “Magna-Graecia” of Catanzaro, 88100 Catanzaro, Italy; 2 Department of Health Sciences, University “Magna-Graecia” of Catanzaro, Viale Europa SNC, 88100 Catanzaro, Italy; 3 Department of Medical and Surgical Sciences, University “Magna-Graecia” of Catanzaro, Viale Europa SNC, 88100 Catanzaro, Italy

**Keywords:** end-stage kidney disease, hemodialysis, peritoneal dialysis, physical activity, METs, depression

## Abstract

Background: Physical inactivity and mood disturbances are key issues in individuals with end-stage kidney disease (ESKD) and may lead to poor clinical outcomes. Methods: We performed a pilot, observational study to explore the possible relationships between the self-reported level of physical activity (IPAQ) and the severity of mood disturbances (BDI score) in a cohort of 58 ESKD patients undergoing chronic hemodialysis (HD; *n* = 30) or peritoneal dialysis (PD; *n* = 28). Results: Overall, ESKD patients were severely inactive (median METs: 590 [460–1850]) and the intensity of overall and walking physical activity was mostly low to moderate. HD individuals appeared less active than PD (METs 550 [250–1600] vs. 1080 [750–1730]; p = 0.003) and were also less prone to walking (METs 180 ± 90 vs. 320 ± 100; p = 0.01), while a barely statistical difference was noticed for the time spent sitting. ESKD individuals displayed a median BDI score of 17 [12–21], which indicated, on average, the presence of borderline depression, which was apparently more evident among HD individuals. A strong, inverse correlation was found between self-reported METs and BDI scores (R = −0.78; *p* < 0.0001), while such scores paralleled the time spent sitting during a weekday (R = 0.45; *p* = 0.0004) and a weekend day (R = 0.40; *p* = 0.002). Conclusions: In ESKD patients on chronic dialysis, physical inactivity and mood disturbances might be significantly inter-connected, thereby amplifying their relative impact on quality of life, dysautonomia and long-term outcomes. Future studies on larger populations are recommended to confirm these preliminary observations. Promoting strategies to improve fitness, along with greater attention to physiological aspects, should be incorporated into the clinical management of ESKD patients.

## 1. Introduction

Patients with end-stage kidney disease (ESKD) require chronic renal replacement therapy (RRT) for their survival, to remove toxins from the body and maintain a normal fluid and electrolyte balance. Currently, there are two different RRT options: hemodialysis (HD), which is an extracorporeal treatment performed for about 4 h/3 times a week in a hospital setting by an artificial kidney machine, and peritoneal dialysis (PD), which is a daily, intracorporeal treatment performed at home with regular timing.

Despite chronic RRT being a life-saving treatment, ESKD patients can remain prone to various complications, exposing them to a high risk of death and poor clinical outcomes.

Muscle atrophy is exceedingly prevalent among dialysis patients and has a multifactorial origin. Malnutrition, which is enhanced by dietary restrictions, sustained protein loss with dialysis treatment and abnormal protein metabolism secondary to inflammation, is a major contributor to muscle wasting and sarcopenia [1,2]. Reduced muscular strength impairs physical capacity, contributing to muscular mass reduction and a lower exercise tolerance [3], thereby establishing a dangerous, vicious circle. Cardiovascular and lung comorbidities, anemia, hypervolemia and tiredness after the dialysis treatment session may worsen such a scenario; as a result, not surprisingly, ESKD patients usually show an extremely sedentary lifestyle, which contributes to their very increased cardiovascular risk.

Mood disturbances, spanning from occasional mood swings to overt clinical depression, are also frequently found among dialysis patients [4]. These arise from a complex interplay of individual predisposition, anxiety, neurohormonal alterations and the eventual presence of cognitive disorders, often manifesting with a variable degree of pessimism, a sense of failure, dissatisfaction and guilt. Lower mood amplifies the negative impact of the disease on quality of life and may finally contribute to the poor clinical outcomes observed in all ESKD populations [4].

With this background in mind, we have carried out a pilot observational study to explore the possible relationships between the effect of physical inactivity and the severity of mood disturbances in a cohort of ESKD patients undergoing chronic RRT by hemodialysis or peritoneal dialysis.

## 2. Materials and Methods

### 2.1. Patients’ Selection

Seventy-one ESKD patients followed at the Nephrology Unit of the “Mater-Domini” University Hospital of Catanzaro, Italy, were screened for eligibility. Of these, 45 were on hemodialysis treatment (HD) and 26 on peritoneal dialysis (PD). Patients on chronic dialysis therapy for less than 6 months, with a history of kidney graft rejection or dialysis modality switching (HD to PD or vice versa) and displaying a severe cognitive impairment or poor clinical condition were excluded from the study.

### 2.2. Physical Activity Self-Assessment

The degree of physical daily activity was assessed by the IPAQ questionnaire (International Physical Activity Questionnaire—Long Form), a 27-item self-reported measure of physical activity, taking into consideration the weekly frequency and average duration (medium–intense) of physical activity, walking activity and the time remaining seated during the day [5]. The IPAQ questionnaire estimates the Metabolic Equivalents of Task (METs) finally delivered. A MET refers to the amount of oxygen consumed while sitting at rest, which is equal to 3.5 mL O_2_ × body weight (kg × min). METs strongly reflect the type of effort made and can therefore be used to compute activities of different intensity together. An estimated weekly MET amount >2520 indicates a good/very good degree of physical activity and an amount of 700–2519 indicates sufficient physical activity, while a weekly METs sum < 700 reflects very limited physical activity with a largely prevalent sedentary habit.

### 2.3. Mood Disturbance Self-Assessment

The presence/severity of mood disturbances was evaluated through the Beck Depression Inventory (BDI) questionnaire [6]. This consists of 21 multiple choice questions considering symptoms such as pessimism, sense of failure, insomnia, self-accusation, self-dissatisfaction and guilt. Depressive symptoms are considered minimal for an overall final score of <10; mild with a score between 10 and 19; moderate with a score between 20 and 29; and serious if the score is >30. Questionnaires were administered by a treating physician before starting a mid-week session in HD patients or before starting a planned outpatient visit in PD patients.

### 2.4. Statistical Analysis

Statistical analysis was performed using the MedCalc software (version 14.8.1; MedCalc Software bvba, Ostend, Belgium) and the GraphPad package (version 9.0.0; GraphPad Software LLC, La Jolla, CA, USA). Data were presented as mean ± SD, median [IQ range] or frequency percentage. Subgroup differences were assessed by an unpaired Student’s *t*-test, a Mann–Whitney test or a Chi-square followed by Fisher’s exact test, as appropriate. Pearson’s correlation coefficient (R) was employed to assess correlations between variables. All results were considered significant for *p* value < 0.05.

## 3. Results

### 3.1. Study Population Characteristics

Of the 71 patients screened, 13 patients (seven HD and six PD) were excluded because they were on dialysis treatment for less than 6 months, were uncooperative or severely disabled or had very poor clinical conditions. The final study cohort thus consisted of 58 ESKD patients (38 HD, 20 PD). The mean age was 64.5 ± 12.3. Patients were predominantly male (62%), with a median time on dialysis of 36 months [range IQ 9–46]. The mean weight at steady state (dry weight) was 62.1 ± 12.3, with a mean BMI of 24 ± 4.3. Moreover, 34% of the patients were diabetic and 72.9% suffered from hypertension. The prevalence of underlying cardiovascular disease ranged from 8.6% (cerebrovascular disease) to 46.5% (ischemic cardiac disease). The history of clinically significant peripheral vascular disease was negligible (5.1%). No statistically significant differences were noticed in the main clinical, anthropometric and laboratory variables between the two subpopulations of HD and PD patients.

Table 1 summarizes the key characteristics of the study population.

### 3.2. Self-Reported Degree of Physical Activity in the Study Population

Results from the IPAQ questionnaire confirmed, on average, a sedentary lifestyle in the study population, with self-reported median METs of 590 [460–1850]. Specifically, only nine patients (15.5%) reported estimated METs > 2520 (good/very good degree of physical activity), while 18 patients (31%) reported total METs of 700–2519 (sufficient physical activity) and 31 patients (53.4%) reported total METs < 700, indicating poor physical activity.

**Figure 1:**
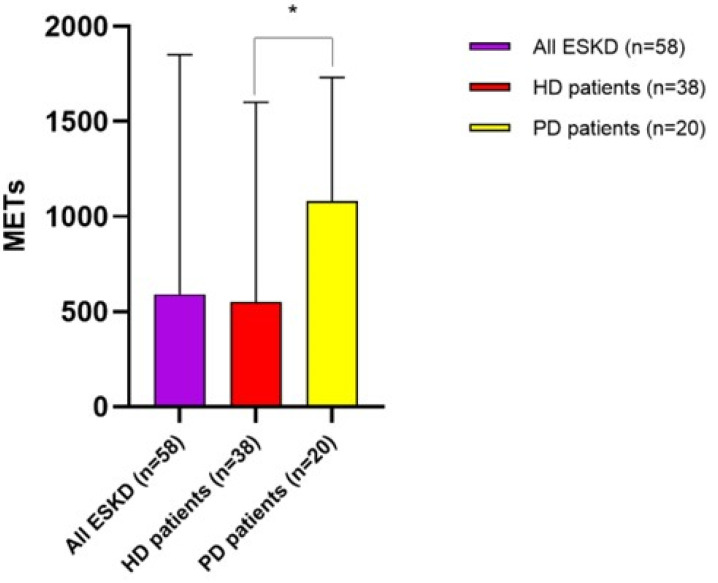
Estimated degree of physical activity (median METs/range) in the whole population and differences between HD and PD patients. * *p* = 0.003.

**Figure 2:**
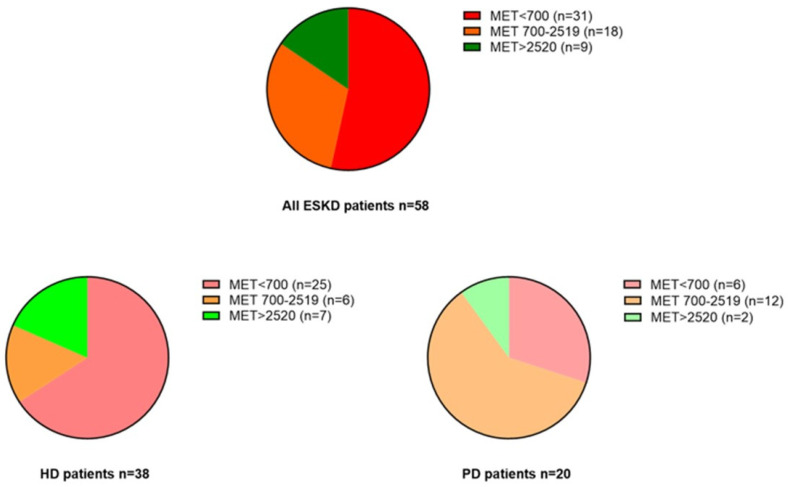
Self-reported intensity of physical activity (METs) in all ESKD patients and in HD and PD patients separately.

Of note, ESKD patients on chronic PD displayed, on average, a significantly better degree of physical activity as compared with those on chronic HD (METs 1080 [750–1730] vs. 550 [250–1600]; p = 0.003). Among these latter, only seven patients (18.4%) reported total METs > 2520, indicating a good/very good degree of physical activity. Of the remaining, 25 patients (65.7%) were largely inactive (total METs < 700), while six patients (15.8%) showed a sufficient degree of physical activity (total METs 700–2519). Among PD patients, two individuals (10%) reported total METs > 2520 and six patients (30%) displayed very low METs < 700), while the remaining 12 (60%) showed intermediate values (total METs 700–2519) (Figure 1 and Figure 2).

Focusing on the intensity of physical activity performed, none of the patients analyzed reported intense activity. The average METs for moderate physical activity in the whole study population were 640 ± 190, but HD patients displayed lower values as compared to PD (420 ± 180 vs. 810 ± 240; *p* = 0.001). Likewise, the average METs from walking were 260 ± 80, but such activity was apparently less practiced by HD patients as compared with PD (180 ± 90 vs. 320 ± 100; *p* = 0.01).

With respect to the intensity of walking, no patient reported intense walking activity, while 50 patients (86.2%) reported slow walking activity (35 patients in HD and 15 in PD) and eight patients (13.8%) moderate walking activity (three patients in HD and five in PD). Finally, the average estimated sitting times during a single working day and during a weekend day were, respectively, 90 ± 25 and 190 ± 90 mins, with no differences noticed between the two subpopulations (Table 2).

### 3.3. Self-Reported Degree of Mood Disturbances in the Study Population

**Figure 3:**
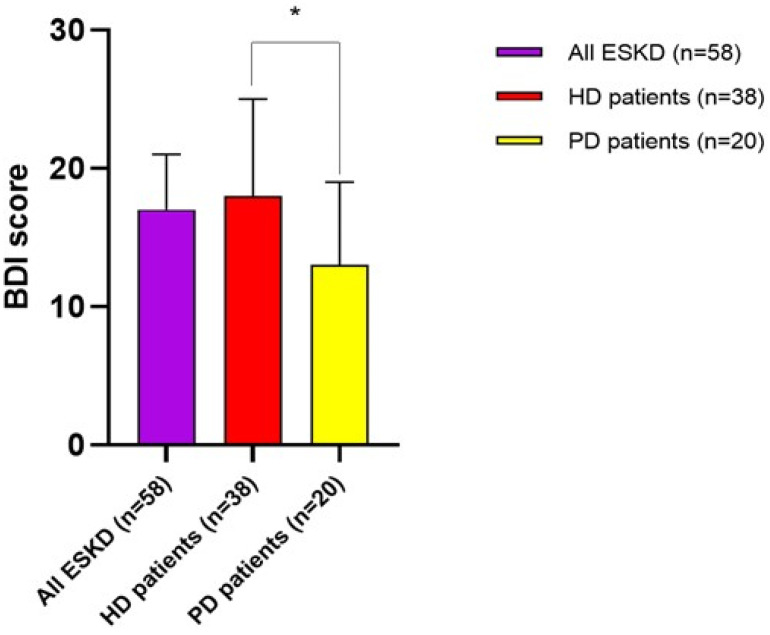
BDI score in all ESKD individuals and differences between HD and PD patients. * *p* = 0.01.

**Figure 4:**
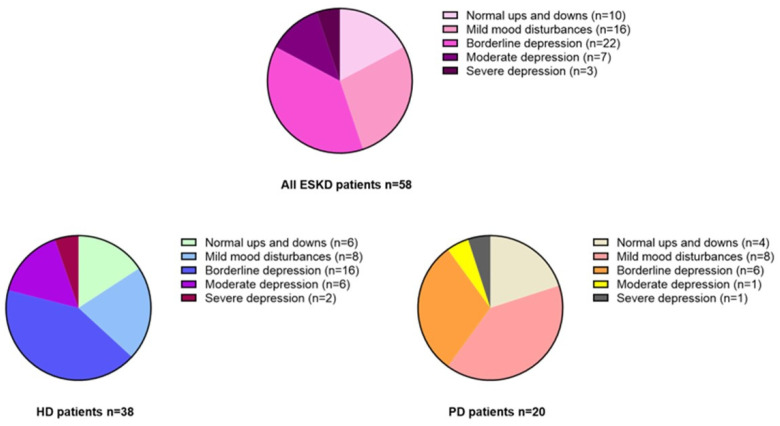
Frequency of self-reported mood disturbances in all ESKD patients and in HD and PD patients separately (BDI score).

ESKD individuals displayed a median BDI score of 17 [12–21], which indicates, on average, the presence of borderline depression in the whole study cohort. Interestingly, HD patients displayed, on average, more severe mood disturbances as compared to PD patients (18 [IQR 11–25] vs. 13 [8–19], respectively; *p* = 0.01). Specifically, 10/58 patients (17.2%, n. 6 in HD and n. 4 in PD) showed occasional mood swings (score 1–10), 16/58 patients (27.6%, n. 8 in HD and n. 8 in PD) mild mood disturbances (score 11–16), 22/58 patients (37.9%, n. 16 in HD and n. 6 in PD) borderline clinical depression (score 17–20), 7/58 (12.1%, n. 6 in HD and n. 1 in PD) moderate depression (score 21–30) and 3/58 (5.2%, *n*. 2 in HD and n. 1 in PD) severe depression (score 31–40) (Figure 3 and Figure 4).

### 3.4. Correlates of Physical Activity and Severity of Mood Disturbances

**Figure 5:**
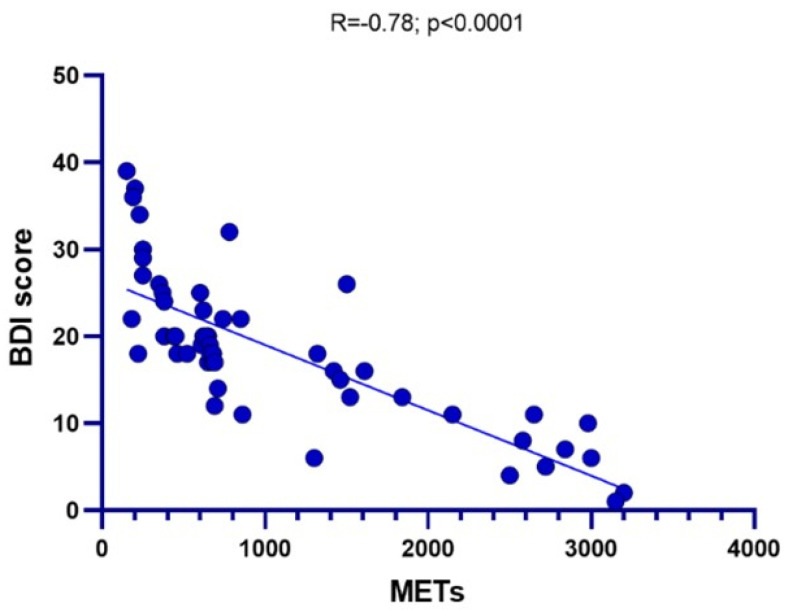
Correlation between BDI score and METs in ESKD patients.

**Figure 6:**
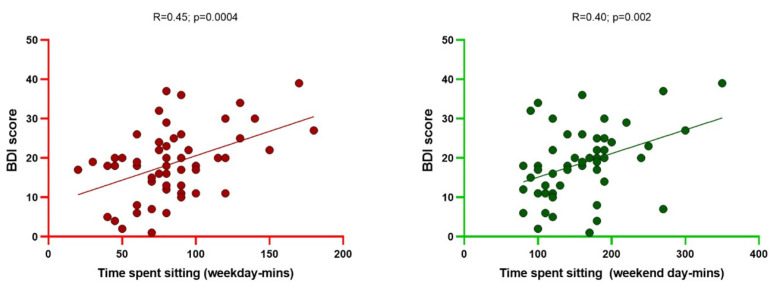
Correlations between BDI score and, respectively, sitting time during a weekday and a weekend day in ESKD patients.

None of the clinical, anthropometric or laboratory variables recorded displayed significant associations with either the estimated degree of physical activity (METs, sitting time) or the severity of depression on the BDI questionnaire (all Pearson R < 0.20; *p* > 0.05). Conversely, a strong, inverse correlation was found between self-reported METs and the BDI score (R = −0.78; *p* < 0.0001). Additionally, such a score was directly related to the number of minutes spent sitting during the week (R = 0.45; *p* = 0.0004), as well as during the weekend (R = 0.40; *p* = 0.002) (Figure 5 and Figure 6).

## 4. Discussion

In our study, we found ESKD patients to be overall inactive, with more than a half of the study population self-reporting a total METs score < 700. Moreover, the physical intensity performed was, on average, light to moderate, and the sitting time on a midweek or weekend day confirmed a propensity towards a sedentary lifestyle.

ESKD persons are notoriously more inactive as compared to healthy people [7,8,9], with muscle wasting, sarcopenia, nutritional deficiency, exercise tolerance and severe chronic inflammation being the main underlying causes [10]. In addition, HD patients usually display lower physical tolerance due to a more enhanced inflammatory state, less controlled anemia, marked arterial hypotension and recurrent fatigue after the hemodialysis sessions [11]. Accordingly, in our study, the overall estimated weekly METs and the specific METs from walking activity were lower in HD patients as compared to PD patients, and the physical activity performed was, on average, of less intensity. Conversely, although evidence exists indicating that HD patients have usually more sedentary behavior than PD [12], we found only a barely significant difference between these two groups in terms of sitting time on a midweek or weekend day, probably due to the very small sample size of the study population.

Another interesting aspect of our study pertained to the observation that ESKD patients self-reported, on average, a state of borderline depression on the Beck questionnaire. Of note, HD patients showed even lower mood levels as compared with individuals on PD; these findings are in line with previous studies confirming that chronic renal replacement therapy, mostly HD, has a significant negative impact on various aspects of psychological health, particularly mental energy and mood [13]. Many aspects may contribute to depression in ESKD patients, including little free time beyond that dedicated to renal therapy, uncertainness and fear of the future, dietary restrictions, social shame and organic cognitive impairment. Nevertheless, in PD individuals, a greater degree of daily autonomy ensuring more physical flexibility, the independence from the dialysis machine, the more physiological treatment and generally better perceived quality of life may somewhat mitigate such negative impacts on daily mood, thus explaining the lower BDI scores reported in this study subpopulation [14,15].

In our opinion, however, the most significant aspect of our study lies in the strong direct correlation found between lower mood and physical inactivity. This was proven by the close inverse relationship between the BDI score and weekly METs and the positive correlation between the degree of depression and time spent sitting.

We were not able to determine whether the reduced physical activity caused/exacerbated the depressive state or vice versa. Predictably, lower individual physical tolerance due to the above-mentioned problems may worsen an already present para-depressive state, which, in turn, may impair socialization, motivation and daily life activities, thereby strengthening the tendency towards solitude and a sedentary lifestyle. Depression negatively impacts virtually all aspects of daily life but can also contribute to poor physical fitness due to a lower desire to perform regular activities and due to the tendency for social isolation. Physical inactivity and mood disturbances might thus be closely connected in ESKD individuals, resulting in amplifying their specific negative impacts on quality of life and patients’ wellbeing.

Physical exercise is nowadays considered a panacea, being a relevant component of the primary and secondary prevention of various cardiovascular, neoplastic, inflammatory and even neurological diseases [16,17]. Additionally, in chronic dialysis patients, regular, low-intensity tailored programs of physical activity are nowadays recommended by various guidelines for the positive influence that they may bring on clinical outcomes, quality of life and even vascular access patency [18,19]. In this regard, the results from our study may further strengthen the importance of a physically active lifestyle, not only to improve fitness and fatigue tolerance, but also to improve mental health, life motivations and social inclusion [20].

Some limitations of our study deserve mentioning. First, the small sample made it difficult to analyze separate correlations between physical activity and depression in the two study subgroups (PD and HD). No less important, the observational pilot nature of the investigation did not allow us to exclude the presence of selection bias and prevented the establishment of causal links between mood disturbances and poor physical functioning. In this view, the implementation of a longitudinal phase could better clarify the temporal relationships and potential causality and, more importantly, could help in uncovering the possible impact of such a correlation on clinical outcomes. Moreover, the lack of a control group of “non-ESKD” individuals may limit the interpretation of the true impact of ESKD itself and the chronic replacement therapy on physical activity and psychological well-being, as compared with the general population.

Finally, the impact of the close relationship between physical inactivity and mood disturbances on other aspects of quality of life deserves appropriate future investigation.

## 5. Conclusions

In conclusion, in this pilot study, we have reported a high degree of physical inactivity and subclinical depression among ESKD patients on chronic renal replacement therapy, particularly in those on HD treatment. Given the pilot, exploratory nature of the study, future investigations on larger and more heterogeneous ESKD populations are recommended to confirm such preliminary findings. Promoting strategies to improve fitness and physical functioning, along with greater attention to physiological aspects, should be incorporated into the clinical management of these particular patients.

## Figures and Tables

**Table 1:** Main clinical characteristics of the whole study population in subgroups of patients undergoing different chronic renal replacement modalities (hemodialysis, HD, and peritoneal dialysis, PD).

	All ESKD (n = 58)	HD Patients (n = 38)	PD Patients (n = 20)	p for Difference
Age (yrs)	64.5 ± 12.3	66.2 ± 8.8	63.1 ± 10.6	0.17
Gender (% male)	62	63.1	60	0.81
Dry weight (kg)	62.1 ± 12.3	65.1 ± 9.9	61.8 ± 13.1	0.65
BMI (Kg/m)	24 ± 4.3	23 ± 3.3	24.8 ± 4.6	0.27
Dialysis vintage (mo.)	36 [9–46]	40 [9–68]	31 [8–33]	0.09
Diabetes (%)	35	26.3	50	0.07
Hypertension (%)	72.4	73.6	70	0.76
Cerebrovascular disease (%)	8.6	10.5	5	0.47
Ischemic cardiac disease (%)	46.5	47.3	40	0.59
Heart failure (%)	22.4	26.1	15	0.32
Peripheral vascular disease (%)	5.1	5.2	5	0.96
Serum phosphate (mg/dL)	5.01 ± 0.86	4.89 ± 0.91	5.12 ± 0.66	0.20
Serum calcium (mg/dL)	9.1 ± 0.77	9.36 ± 0.55	8.81 ± 0.87	0.18
Serum potassium (mg/dL)	4.81 ± 0.54	4.59 ± 0.78	5.33 ± 0.81	0.07
Serum sodium (mg/dL)	139.3 ± 3.2	140.6 ± 7.5	138.8 ± 8.7	0.54
Parathormone (pg/mL)	236.9 [147.8–505.6]	296.3 [87.6–303.6]	188.4 [50.8–205.3]	0.66
Albumin (g/dL)	3.99 ± 0.79	3.66 ± 0.98	4.12 ± 0.86	0.32
Total cholesterol (mg/dL)	160.1 ± 41.3	164.5 ± 62.5	160.5 ± 38.3	0.40
Triglycerides (mg/dL)	139.3 ± 68.9	144.1 ± 70	138.5 ± 45.2	0.21
Creatinine (mg/dL)	9.2 [8.6–12.2]	8.6 ± 4.2	10.1 [5.5–11.3]	0.47
Hematocrit (%)	35.2 ± 6.1	34.2 ± 5.9	37.1 ± 5.8	0.32
Hemoglobin (g/dL)	11.0 ± 0.7	10.9 ± 0.6	11.0 ± 0.7	0.89
Ferritin (mg/dL)	203.1 [65.4–312.5]	233.6 [195.1–310.2]	188.1 [44.8–195.5]	0.23
Serum iron (mg/dL)	60.2 [31.5–92.8]	59.1 [38.8–66.6]	61.3 [48.2–80.3]	0.31
Urea (mg/dL)	144.8 ± 38.3	148.3 ± 40.1	141.1 ± 38.1	0.08

**Table 2:** Estimated METs according to the self-reported intensity and degree of daily physical activity and time spent sitting in the whole ESKD population and differences between HD and PD patients. Statistical differences are highlighted in bold.

Activity Indicators	Total	HD	PD	*p*
METs intense activity	0	0	0	-
METs moderate activity	640 ± 190	420 ± 180	810 ± 240	**0.001**
METs walking	260 ± 80	180 ± 90	320 ± 100	**0.01**
Total METs	590 [460–1850]	550 [250–1600]	1080 [750–1730]	**0.003**
Sitting time weekday (min)	90 ± 25	95 ± 40	88 ± 40	0.17
Sitting time weekend day (min)	190 ± 90	200 ± 80	190 ± 70	0.09

## Data Availability

Raw data from the present study are available from the Corresponding Author upon reasonable request.
